# Detection of African swine fever virus in pork products brought to Taiwan by travellers

**DOI:** 10.1080/22221751.2019.1636615

**Published:** 2019-07-03

**Authors:** Wen-Hung Wang, Chih-Yen Lin, Max R. Chang Ishcol, Aspiro Nayim Urbina, Wanchai Assavalapsakul, Arunee Thitithanyanont, Po-Liang Lu, Yen-Hsu Chen, Sheng-Fan Wang

**Affiliations:** a Division of Infectious Disease, Department of Internal Medicine, Kaohsiung Medical University Hospital, Kaohsiung Medical University, Kaohsiung, Taiwan; b Center for Tropical Medicine and Infectious Disease, Kaohsiung Medical University, Kaohsiung, Taiwan; c Department of Medical Laboratory Science and Biotechnology, Kaohsiung Medical University, Kaohsiung, Taiwan; d Program in Tropical Medicine, College of Medicine, Kaohsiung Medical University, Kaohsiung City, Taiwan; e Department of Microbiology, Faculty of Science, Chulalongkorn University, Bangkok, Thailand; f Department of Microbiology, Faculty of Science, Mahidol University, Bangkok, Thailand; g Department of Medical Research, Kaohsiung Medical University Hospital, Kaohsiung, Taiwan

African swine fever (ASF) is a highly contagious hemorrhagic viral disease associated with high fatality rates in domestic and wild pigs. It is caused by the African swine fever virus (ASFV) [1]. ASFV is a double stranded DNA virus and is the only member of the family *Asfarvidae*, genus *Asfivirus*; and is known to infect warthogs and ticks without causing disease [1]. Historically, ASF was first reported in 1921 infecting domestic pigs from Kenya, Africa. In recent years, early 2017, an ASF outbreak was reported in Irkutsk, far eastern Russia [1,2]. Thereafter, outbreaks near the borders of China were reported, demonstrating that ASFV has a remarkable capacity for transboundary and transcontinental spread [3,4] (S-Figure 1). The first ASF outbreak in China was reported from a farm near Shenyang City in Liaoning province in June, 2018 [5]. Since then, distribution of ASF has been rapidly spreading, resulting in several outbreaks on different provinces in China [3,6]. According to World Organization for Animal Health (OIE), up until 12-June 2019, there were 143 cases of laboratory confirmed ASFV infected domestic or wild pigs reported throughout 32 provinces in China [7]. Due to the high sequence similarity of the Chinese isolates with several strains from Eastern Europe and Africa, the source of ASFV transmitted to China remains unclear [6] (S-Figure 1). Of note, it is worthy to mention that currently there is still a lack of vaccine and antiviral drugs against ASFV infection [1,8].

Taiwan is an island located in the South China Sea and is of close proximity to Mainland China. The main island of Taiwan is surrounded by other smaller islands with the larger ones including Matsu, Kinmen, Penghu, Green, and Orchid Islands. In recent years, the frequency and number of economic activities such as, trading and business travelling, have increased between China and Taiwan. Due to the fact that over 5.4 million pigs are raised in Taiwan, local pig Farmers are concern of the spread of ASF from neighbouring countries such Vietnam, China, Mongolia and Korea where ASFV-infected pigs have been confirmed [9]. Although ASF has never been reported in Taiwan, ASFVs may easily be introduced into this country through various routes. The risk of introducing ASF into Taiwan might intensification with the introduction of ASFVs-contaminated pork products brought by travellers, as well as, imported ASFV-infected pigs. Pork products contaminated with ASFV are currently the main risk factors for disseminating the disease. The Bureau of Animal and Plant Health inspection and Quarantine (BAPHIQ) announced the first finding of ASFVs detected in sausages coming from China, carried by travellers going to Kinmen in 31-October 2018 [9]. Later in 31-December of 2018, a dead pig washed ashore on Kinmen Island. This dead pig was confirmed to be a ASF positive case possessing the virus belonging to genotype II, which has been the predominant strain circulating in Asian and European countries in recent years [9].

Hence, Taiwan has initiated strict surveillance on pork products confiscated at airports and ports from travellers coming from countries affected by ASF since October-2018. Up until May-2019, there has been a total of 1667 pork products including sausage, ham, pork jerky and grilled pork confiscated by different airports or ports of Taiwan (Figure 1A). These confiscated products have been sent to BAPHIQ for ASFV testing using TaqMan qRT-PCR (Applied Biosystems) used to amplify the partial conserved regions of the ASFV *B646L(VP72)* gene as recommended by OIE [10]. The primers and probes used for the detection of ASFV are listed as: ASFV-F-5’-CTGCTCATGGTATCAATCTTATCGA-3’(Forward); ASFV-R-5’-GATACCACAAGATCRGCCGT-3’(Reverse) and ASFV-Probe: FAM-5’-CCACGGGAGGAATACCAACCCAGTG-3’-TAMRA. Results revealed that there were 62 ASFV confirmed cases. A total of 59 ASFV positive pork products carried by travellers were from different provinces of China and 3 ASFV positive products were from Vietnam (Figure 1B&C). The average po detected sitive rate was 3.7% (Figure 1B). In addition, Taiwanese authorities encouraged the travellers to report importing pork products actively to the quarantine counter or discard the pork products into the disposal box by themselves. Data from the BAPHIQ indicated that the daily average of disposed meat increased from 20 kg to over 60 kg between December-2018 to Janurary-2019 and showed a decreasing after Feburary-2019 (Figure 1D), suggesting that this policy might be efficiently blocking ASFV transmission to Taiwan via disposal of pork products from ASF outbreak countries. Data from ASFV suspected pigs indicates that there were 168 sick pigs sent for screening; however, all the pigs were identified as ASFV(-) (Figure 1E). The severity of the ASF epidemics has worsen in China [7]. Recently, the Food and Agriculture Organization of the United Nations (FAO) announced the high possibility of ASFV spreading from China to other countries of Asia [11]. More recently, a report revealed the presence of ASFV in pork products brought into South Korea by travellers from China [12]. Such findings demonstrates the role of travellers as being a major risk factor and pose a potential threat by bringing pork products from outbreak areas [12]. In addition, kitchen scraps containing pork contaminated with ASFVs used for pig feeding was reported to be a potential source correlated with persistent ASF outbreaks in these epidemic countries [3,7].

**Figure 1.**
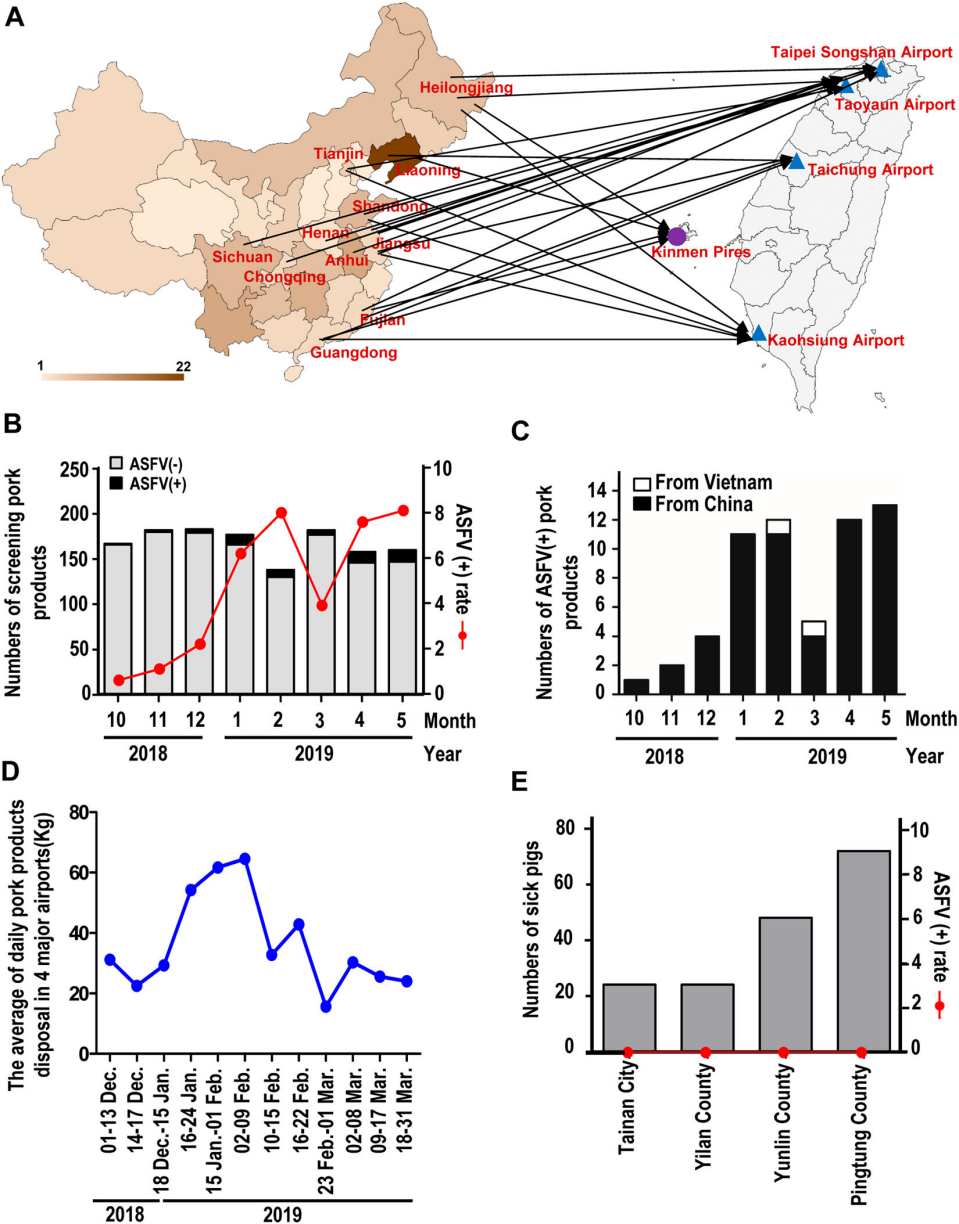
African swine fever virus transmitted by contaminated pork products brought by travellers. (A) The ASFV positive pork products from different provinces of China collected in the entry sites including airports and ports of Taiwan. (B) Number of monthly ASFV positive pork products brought by travellers detected in Taiwan. (C) The sources of ASFV positive pork products. (D)The average weight of pork products disposal by travellers. (E) The diagnosis and screening of suspected ASFV infected pigs in Taiwan.

Although ASFVs does not cause disease in humans, it is regarded as a highly contagious agents causing high mortality in domestic pigs; leading to severe economic loss. To prevent any chance of laboratory leaks or dissemination, all the diagnosis and virus isolation of ASFVs are handled only by BAPHIQ. Other medical centres and virological laboratories are not authorized to conduct this diagnosis. Due to the policy of immediate slaughter and clearance of suspected ASFVs infected pigs, and proper disposal of pork products there have been no isolated ASFVs cases. In addition, Real-time PCR amplified *VP72* gene segments from most circulated ASFV genotype II isolates were reported to be in a conserved and stable cluster under phylogenetic tree analysis (Bootstrap value >90) [6,13]. The source of ASFV detected from the dead pig and pork products in Taiwan needs to be confirmed by checking the similarity using virus isolation and whole virus genome sequencing.

In summary, this study reports the current situation regarding ASFV detection in pork products brought by travellers to Taiwan and the policies against ASFV outbreak in Taiwan. Travellers that privately bring pork products from ASFV affected countries have been considered as a potential threat to introduce and spread ASFV in an uninfected area. Strict border inspections of confiscated products are urgently required to control and prevention of ASFV attacking Taiwan and consequent dissemination to other Asian countries.

## Supplementary Material

Supplemental Material
