# Supplementary material for: Detection of African swine fever virus in pork products brought to Taiwan by travellers
Source: Emerg Microbes Infect. 2019 Jul 3;8(1):1000–2. doi: 10.1080/22221751.2019.1636615 (PMC6609336; doi:10.1080/22221751.2019.1636615)

**Supplementary data:**

**FIGURE LEGENDS:**

**S-Fig.1 The history of African swine fever transmission.**

The first African swine fever (ASF) was reported from Kenya, African in 1921. After that ASF has been transmitted to other areas including Europe, Latin American, Russia and China.


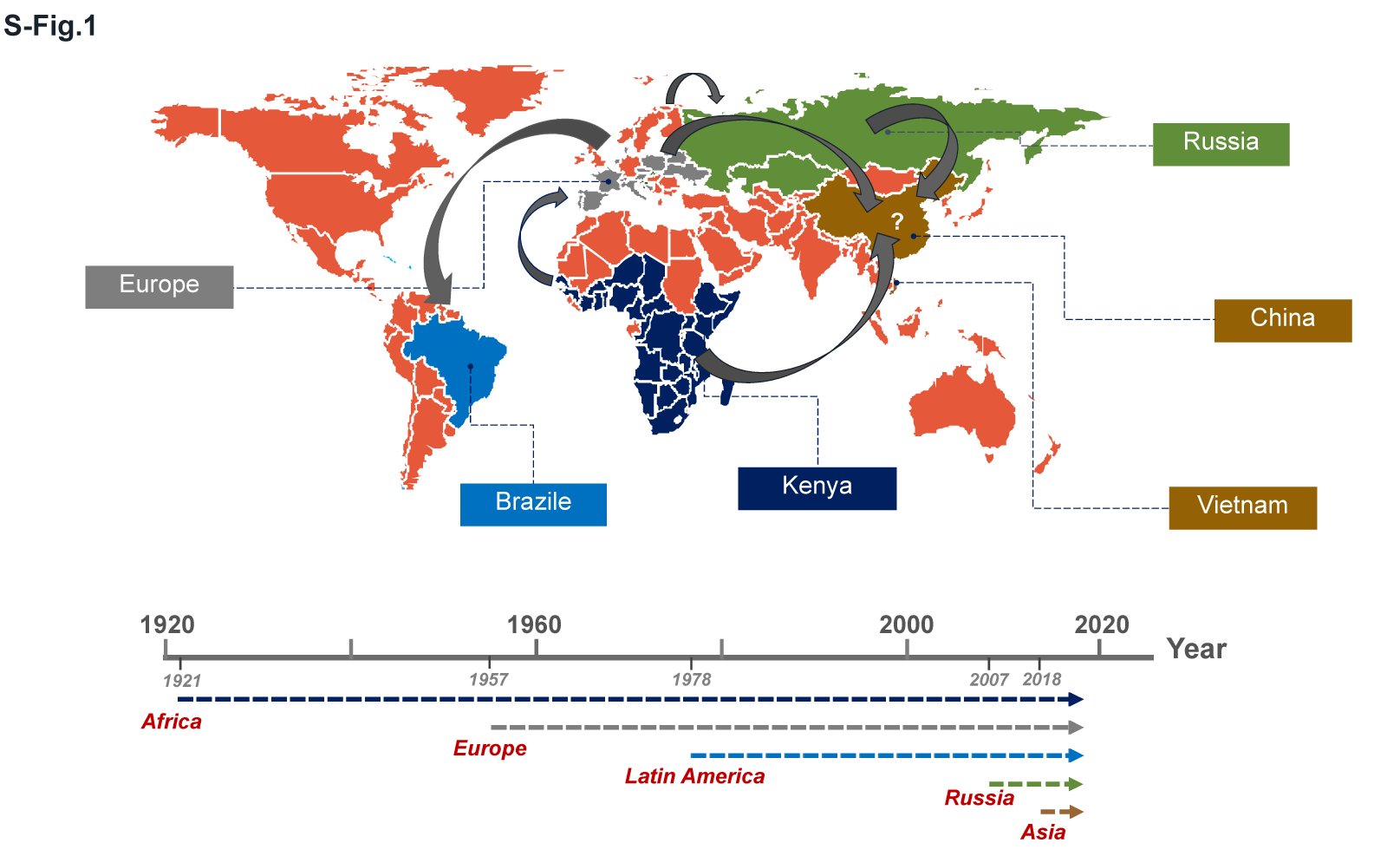

Supplement: Supplemental Material [file TEMI_A_1636615_SM4705.doc]
